# When the Flu Breaks the Heart: A Case Report of Influenza A-Induced Takotsubo Cardiomyopathy

**DOI:** 10.7759/cureus.113390

**Published:** 2026-07-26

**Authors:** Immad Attique, Alex Gyftopoulos, Deya Alkhatib, Raiha Shahbaz

**Affiliations:** 1 Internal Medicine, Bayhealth Hospital, Milford, USA; 2 Cardiology, University of Maryland School of Medicine, Baltimore, USA; 3 Cardiology, Medical College of Georgia, Augusta University, Augusta, USA; 4 Internal Medicine, Nishtar Medical University, Multan, PAK

**Keywords:** broken-heart syndrome, influenza a infection, influenza complication, stress induced cardiomyopathy, takotsubo cardiomyopathy (ttc)

## Abstract

We present a case of Takotsubo cardiomyopathy (TCM), commonly known as broken heart syndrome, triggered by influenza A infection. Known cardiac complications of influenza include myocardial infarction, myocarditis, and pericarditis. Only a few cases of influenza have so far been reported that led to TCM. We discuss the presentation, investigations, and management of a 67-year-old woman who presented to the emergency department (ED) with chest pain and shortness of breath two weeks after being diagnosed with influenza A. We aim to highlight a rare cardiac complication of influenza infection so that clinicians can have a high index of suspicion for this association.

## Introduction

Takotsubo cardiomyopathy (TCM), also known as stress-induced cardiomyopathy, presents as an acute coronary syndrome (ACS) characterized by severe left ventricular (LV) dysfunction associated with emotional, psychological, or physiological stressors, including infectious causes [1,2]. To date, TCM has only rarely been associated with influenza infection [3-6]. Influenza more commonly exacerbates underlying heart problems such as congestive heart failure or ischemic heart disease [7]. Most of the reported cases of TCM associated with influenza infection were associated with symptom onset either at the time of or within two to five days after being diagnosed with influenza [4-6]. Our case is different in that the patient developed symptoms after two weeks of being diagnosed with influenza A infection.

## Case presentation

A 67-year-old woman initially presented to the emergency department (ED) with complaints of cough, body aches, and back pain for the last two days. She also reported associated chest pain, nausea, vomiting, and diarrhea. Upon physical examination, she was afebrile, normotensive, and saturating 97% on room air. Remarkable exam findings included decreased breath sounds and bilateral flank tenderness. A 12-lead electrocardiogram (ECG) was negative for any acute ischemic changes. A rapid influenza A test was positive, and she was discharged home with oseltamivir. Two weeks later, she experienced three episodes of squeezing chest pain associated with shortness of breath that were triggered by walking. Her symptoms resolved with rest. While being evaluated in the ED, she complained of sudden-onset chest pain that was radiating to her left arm. She denied palpitations, cough, or sputum production. She denied a history of myocardial infarction. Her family history is significant for hypertension in her mother. Her social history is relevant for a remote history of intravenous drug use 14 years prior. She smokes one-fifth of a pack of cigarettes per day and has two alcoholic drinks per week. On examination, she appeared in acute distress due to pain. Her vitals included a forehead temperature of 98.2°F, pulse rate of 75 beats per minute, blood pressure of 135/70, respiratory rate of 19, and oxygen saturation of 99% on room air.

Past medical history

The patient’s past medical history includes hypertension, diabetes mellitus, anxiety, depression, bipolar disorder, and post-menopausal status.

Differential diagnosis

Differential diagnosis for a patient presenting with chest pain in the setting of recent viral respiratory tract infection includes ACS, such as unstable angina, non-ST-segment myocardial infarction, or ST-segment myocardial infarction, myocarditis, pericarditis, and TCM. Other differentials to consider include aortic dissection, acute pulmonary edema, pulmonary embolism, pneumonia, gastritis, and mesenteric ischemia.

Investigations and management

**Figure 1:**
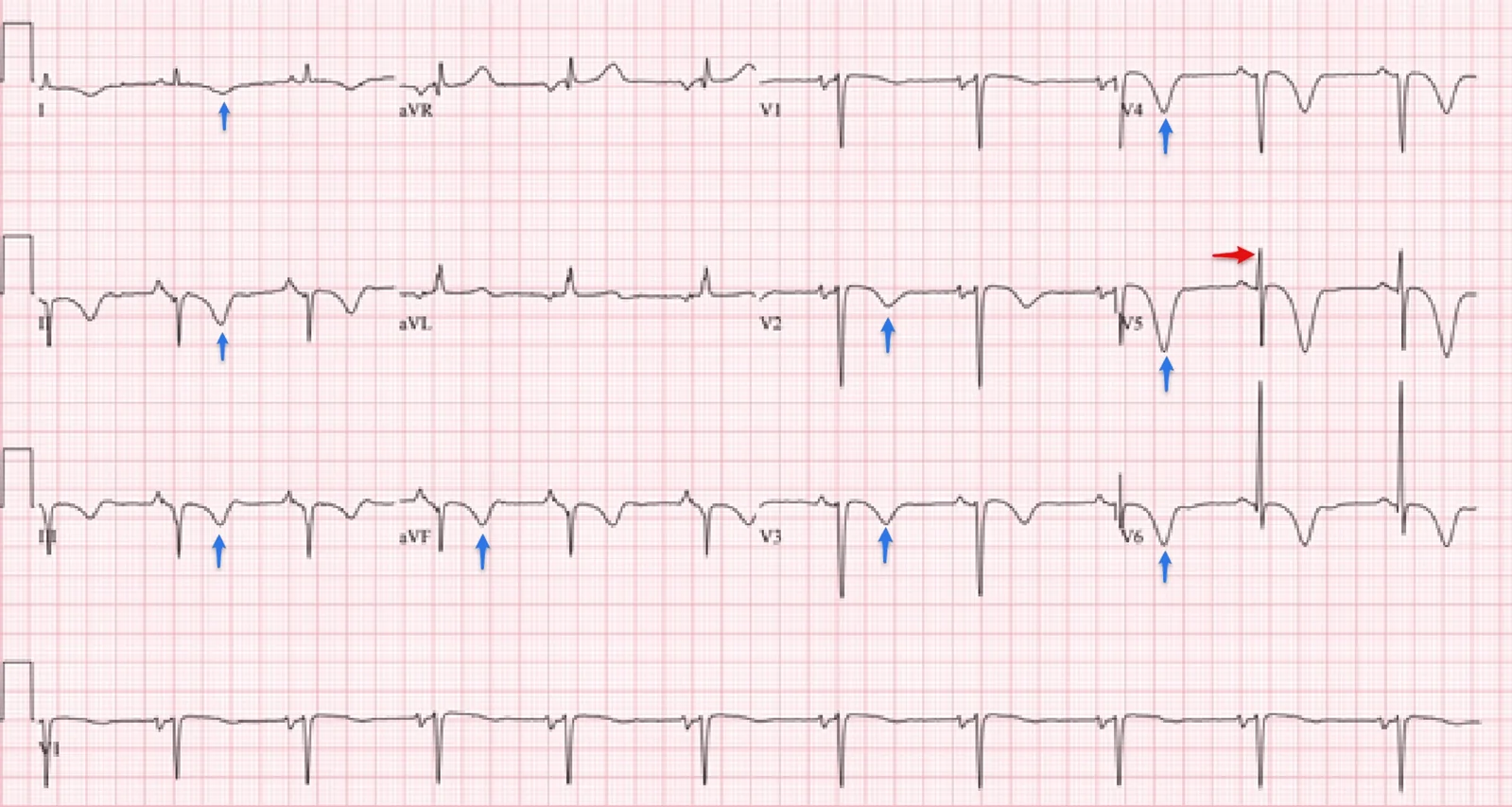
Initial 12-lead electrocardiogram showing T-wave inversion (blue arrow) and poor R wave (red arrow) progression from V1-V4

Figure 1 shows the initial 12-lead ECG obtained. It exhibits T-wave inversion in the inferior leads (II, III, aVF), the precordial leads V2-V6, and lead I, along with poor R-wave progression in the precordial leads V1-V4.

Chest radiograph showed no acute cardiopulmonary abnormality. Chest computed tomography angiography (CTA) was negative for aortic dissection, pulmonary embolism, and significant pericardial effusion. It showed atherosclerotic mural calcification of the abdominal aorta, with chronic occlusion of the celiac artery and superior mesenteric artery (SMA) with distal reconstitution from inferior mesenteric artery (IMA) collaterals.

Pertinent laboratory findings at admission are described in Table 1.

**Table 1:** Pertinent laboratory findings at admission

Parameter	Patient Value	Normal Adult Reference Range
Troponin-I	0.6 ng/mL	0-0.04 ng/mL
Aspartate aminotransferase (AST)	41 U/L	0-35 U/L
Alanine aminotransferase (ALT)	19 U/L	4-42 U/L
Total bilirubin	0.6 mg/dL	0.3-1.0 mg/dL
Alkaline phosphatase (ALP)	83 U/L	30-120 U/L

Rapid influenza test was positive for influenza A. After ruling out aortic dissection, the patient was loaded with clopidogrel 600 mg and aspirin 325 mg. She was started on intravenous heparin per ACS protocol. Troponin-I peaked at 1.4 ng/mL approximately five hours after the initial blood draw.

A transthoracic echocardiogram (TTE) showed normal LV size with severely reduced LV systolic function (LV ejection fraction of 30%), and almost global hypokinesis with sparing of the basal inferolateral wall.

Video 1 shows TTE apical four-chamber view.

**Video 1:** Transthoracic echocardiography (TTE) apical four-chamber view TTE apical four-chamber view depicting left ventricular systolic dysfunction with anterolateral and apical hypokinesis. Left ventricular ejection fraction visually estimated at 30%.

The patient underwent cardiac catheterization, which demonstrated non-obstructive coronary artery disease. Left ventriculography revealed severe apical hypokinesis and hyperdynamic basal segments consistent with stress-induced TCM.

Following catheterization, she was started on a statin and an angiotensin-converting enzyme (ACE) inhibitor and discharged with prompt follow-up with cardiology.

## Discussion

Influenza infection affected 39 to 56 million people in the United States (U.S.) during the 2019-2020 U.S. flu season [8]. Pulmonary manifestations of influenza are most common. Neuromuscular and cardiac complications are unusual but may manifest in persons of any age [7]. Cardiac complications of influenza include myocarditis, pericarditis, and acute myocardial infarction [7,9]. It has only been implicated in a few case reports as the cause of TCM [3-6].

TCM, also called “broken heart” syndrome or apical ballooning syndrome, is a reversible cardiomyopathy characterized by LV dysfunction and ballooning of the LV apex on imaging during systole [10]. TCM was first described in Japan in 1990. It was named Takotsubo because the shape of the left ventricle resembles a Japanese octopus tray, with a round bottom and narrow neck [11]. Our patient’s past medical history included anxiety and post-menopausal state, both of which are risk factors for the development of TCM. Other risk factors include age >55 years, smoking history, alcohol abuse, and hyperlipidemia [10,12]. The pathophysiology of TCM is not fully understood. Possible mechanisms include increased concentration of catecholamines as a result of emotional and/or physical stressors, which can lead to cardiac sympathetic hyperactivity, causing direct myocardial injury, coronary microvascular spasm and/or vasoconstriction, and increased cardiac workload resulting in supply-demand mismatch and postischemic stunning [1]. Patients with TCM most commonly present with chest pain and dyspnea. Common ECG changes can include ST-segment elevation, T-wave inversion, or prolongation of the QT interval. Cardiac biomarkers are only mildly elevated [1,11]. The most common diagnostic criteria used for TCM are “Modified Mayo Clinic Criteria (2008) for Apical Ballooning Syndrome/Takotsubo Cardiomyopathy,” which is given in Table 2 [13]. Other criteria that can be used for diagnosis are Heart Failure Association of the European Society of Cardiology (HFA-ESC) diagnostic criteria for Takotsubo syndrome [14] and the International Takotsubo Diagnostic Criteria (InterTAK Diagnostic Criteria) [15].

**Table 2:** Mayo Clinic Criteria for ABS/TTC The table lists the Mayo Clinic Criteria for ABS/TTC, reproduced from [13] under the terms of the Creative Commons Attribution-NonCommercial-NoDerivatives 4.0 International License (CC BY-NC-ND 4.0). * There are rare exceptions to these criteria, such as those patients in whom the regional wall motion abnormality is limited to a single coronary territory. ^†^ It is possible that a patient with obstructive coronary atherosclerosis may also develop ABS. However, this is very rare in our experience and in the published literature, perhaps because such cases are misdiagnosed as ACS. In both of the above circumstances, the diagnosis of ABS should be made with caution and a clear stressful precipitating trigger must be sought. ABS: apical ballooning syndrome; TTC: Takotsubo cardiomyopathy; ACS: acute coronary syndrome

Mayo Clinic Criteria for ABS/TTC
1. Transient hypokinesis, akinesis, or dyskinesis of the left ventricular mid-segments with or without apical involvement; the regional wall motion abnormalities extend beyond a single epicardial vascular distribution; a stressful trigger is often, but not always, present.*
2. Absence of obstructive coronary disease or angiographic evidence of acute plaque rupture.^†^
3. New electrocardiographic abnormalities (either ST-segment elevation and/or T-wave inversion) or modest elevation in cardiac troponin.
4. Absence of:
a. Pheochromocytoma
b. Myocarditis

Initial treatment of patients with suspected TCM includes supplemental oxygen if needed, intravenous heparin, aspirin, and beta blockers, as it mimics acute myocardial ischemia. Once the diagnosis of TCM is confirmed, aspirin can be discontinued. It is reasonable to use ACE inhibitors or angiotensin II type 1 receptor blockers during the period when regional wall motion abnormality is present. Systemic anticoagulation therapy should be continued until resolution of regional wall motion abnormality in the apical segment in order to prevent LV apical thrombosis. Complications of TCM include congestive heart failure, cardiogenic shock, LV apical thrombosis, arrhythmias, and LV free wall rupture or septal perforation [16]. TCM has an excellent prognosis. Complete recovery of LV function occurs in three to four weeks. In-hospital mortality rate varies from 1.1% to 2%. Recurrence rate ranges from 0% to 15% [11,16].

## Conclusions

TCM can be a rare complication of influenza infection. Clinicians should maintain a high index of suspicion for TCM in patients presenting with signs and symptoms of ACS, particularly in the setting of a recent influenza infection. Early recognition of this condition is essential for appropriate evaluation and management. Clinicians should encourage their patients to get vaccinated for influenza, as this is an important preventive measure to reduce the risk of influenza infection and its potential complications, including TCM.
